# Comparison of self-assessed and clinician-assessed hirsutism diagnosed according to the modified Ferriman-Gallwey scale among female outpatients in Brazil

**DOI:** 10.20945/2359-4292-2023-0271

**Published:** 2024-06-24

**Authors:** Talita Fernanda Oliveira, Talita Fischer Oliveira, Dayane Campos Santana, Ana Luiza Lunardi Rocha, Ana Lucia Cândido, Fabio Vasconcellos Comim

**Affiliations:** 1 Departamento de Clínica Médica Faculdade de Medicina Universidade Federal de Minas Gerais Belo Horizonte MG Brasil Departamento de Clínica Médica, Faculdade de Medicina, Universidade Federal de Minas Gerais, Belo Horizonte, MG, Brasil; 2 Departamento de Ginecologia Faculdade de Medicina Universidade Federal de Minas Gerais Belo Horizonte MG Brasil Departamento de Ginecologia, Faculdade de Medicina, Universidade Federal de Minas Gerais, Belo Horizonte, MG, Brasil; 3 Faculdade de Ciências Médicas de Minas Gerais Belo Horizonte MG Brasil Faculdade de Ciências Médicas de Minas Gerais, Belo Horizonte, MG, Brasil

**Keywords:** Hirsutism, self-assessment, assessment, process, hyperandrogenism, polycystic ovary syndrome

## Abstract

**Objective:**

The aim of this study was to evaluate the efficacy of a self-assessment questionnaire for hirsutism using the latest cutoff values recommended by the Endocrine Society (ES) for Latin-American women and by the European Society for Human Reproduction and Embryology (ESHRE).

**Subject and methods:**

Female premenopausal outpatients (n = 188) completed a self-assessment questionnaire, scoring hair presence across the nine areas evaluated by the modified Ferriman-Gallwey (mFG) scale. The results were compared with clinician-assessed scores rated independently by two trained physicians. Scores in the Hirsuta questionnaire, derived from self-assessment of five areas of the mFG scale, were also evaluated.

**Results:**

The ethnic composition of the sample was as follows: 23.1% white, 25.8% black, 48.9% mixed, and 2.1% other backgrounds (Indigenous, Asian). The participants had age and BMI of (mean ± standard deviation) 33.7 ± 9.9 years and 29.8 ± 7.21 kg/m^2^, respectively. The most common areas of excessive hair growth were the chin, upper and lower abdomen, and thighs. Relative to clinician-assessed mFG scores, self-assessed mFG scores had an accuracy of 80% using ES criteria for hirsutism diagnosis, with a sensitivity of 95.45%, specificity of 56.25%, positive predictive value of 30.10%, and negative predictive value of 98.40%. Self-assessed mFG had lower accuracy (71%) for diagnosing hirsutism when the ESHRE criteria were applied.

**Conclusions:**

Self-assessed mFG had low specificity, limiting its application. The results of this study do not support the use of the self-assessed mFG or Hirsuta scores for diagnosing hirsutism in a clinical setting, although both scoring systems may be useful for screening hirsutism in epidemiological studies.

## INTRODUCTION

Hirsutism is a common condition that affects around 10%-15% of women and is characterized by excessive growth of terminal hair distributed in a pattern typically observed in men (1-3). Clinical diagnosis of hirsutism is required for the identification of key hyperandrogenic disorders such as polycystic ovary syndrome and virilizing tumors (3,4). Even when not associated with other comorbidities, hirsutism has a negative impact on women’s quality of life (5).

Currently, the gold-standard method for diagnosing hirsutism is the modified Ferriman-Gallwey scale (mFG). This system evaluates hair growth across nine body areas (upper lip, chin, arms, upper and lower abdomen, chest, legs, and upper and lower back) (1,6). The cutoff values for hirsutism based on the mFG score vary by ethnicity. Thus, scores ≥ 8 identify hirsutism in Caucasian women while scores ≥ 6 identify hirsutism in Latin-American women (3,6). Notably, the evaluation of the mFG score requires time, good illumination, and a trained investigator (7-9).

Because of intrinsic difficulties in determining the presence of hirsutism, other approaches to establish the diagnosis of excessive hair growth have been proposed, in particular, the self-assessed mFG scores (2,7,10-13). Results from these studies in different populations have shown variable correlations between self-assessed and clinician-assessed mFG (7).

Opinions regarding self-assessment of hirsutism vary among authors. While some caution against self-assessment due to the potential for misclassification or overestimation of hirsutism’s severity (2,7), others acknowledge the usefulness of self-assessment to a limited or moderate degree (10-12). One study demonstrated 89% accuracy and internal consistency with a self-assessment questionnaire for identifying hirsutism among 90 women in Brazil (the Hirsuta score) (11). The participants used a self-administered instrument to evaluate hair distribution across five body areas, and their responses were verified against the mFG (11).

In light of these observations, our study evaluated the accuracy of the self-assessed mFG using a structured questionnaire and the Hirsuta score against mFG scores independently rated by two blinded trained observers (clinician-assessed mFG). The results were analyzed considering cutoff values recommended by the Endocrine Society (ES) for Latin-American women and by the European Society for Human Reproduction and Embryology (ESHRE).

## SUBJECTS AND METHODS

This cross-sectional study was approved by the ethics committee of Federal University of Minas Gerais (UFMG), Brazil (CAAE 30675320.4.0000.5149). All participants signed an informed consent form.

### Patients

Women aged 18-50 years were recruited from endocrine and gynecologic outpatient clinics at *Hospital das Clínicas* at UFMG from July 2021 to December 2022. All women with this condition were included, even those using medications or being treated for chronic disorders. The exclusion criteria were the presence of any of the following conditions: (A) generalized skin disease (*e.g.*, pemphigus, cellulitis, erythroderma), cosmetic treatment (*e.g.*, tattoos), or any other situation (*e.g.*, use of bandage) that could impair the correct visualization of more than one area assessed in the mFG score; (B) androgen treatment (*e.g.*, female-to-male transgender people or other similar situations); (C) use of medications that accelerate hair growth (*e.g.*, phenytoin, acetazolamide, cyclosporine, minoxidil, streptomycin, psoralen); and (D) any psychiatric conditions that may affect mFG scoring.

The participants underwent clinical evaluation that included data collection about history of alopecia, hirsutism, menstrual cycles, use of medications, family history of hirsutism, and complaints about excessive hair growth. Anthropometric measurements were obtained, including body mass index (BMI) and abdominal circumference. Two blinded independent clinicians assessed the participants’ mFG scores under optimal conditions. The clinicians were previously trained and demonstrated a high level of inter-rater reliability with a kappa coefficient above 0.9. Information about recent history of hair removal or discoloration was obtained for each area assessed in the mFG score.

To establish the diagnosis of hirsutism, we adopted the latest criteria recommended by the Endocrine Society (ES) and the European Society for Human Reproduction and Embryology (ESHRE). These criteria consider the diagnosis of hirsutism at cutoff values ≥ 6 in Latin-American women and ≥ 4, respectively.

The participants were asked to complete a questionnaire with detailed illustrations of increasing hair growth degrees in the nine body areas assessed by the mFG score, rating their scores for each area (Supplementary Figure 1). To prevent misclassification of hirsutism, we excluded from the analysis women who had one or more areas of hair removed (41 and 44 women in the ES and ESHRE analysis, respectively).

**Figure 1:**
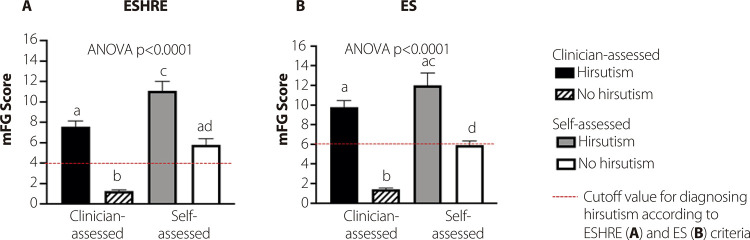
Mean clinician-assessed and self-assessed modified Ferriman-Gallwey (mFG) scores considering the criteria proposed by the (A) European Society for Human Reproduction and Embryology (ESHRE) and (B) Endocrine Society. Abbreviation: ANOVA, analysis of variance.

### Statistical analysis

The software Jamovi, Version 2.2 (http://www.jamovi.org) was utilized for statistical analysis, with all data being anonymized before input. The normality of the variables’ distributions was verified using the Shapiro-Wilk test. Student’s *t* test was applied for data with normal distribution, and the Mann-Whitney U test was utilized for data with non-normal distribution. Fisher’s exact test was employed to compare categorical data. P values < 0.05 were considered statistically significant. The Bland-Altman analysis was performed to compare the two approaches for hirsutism diagnosis (*i.e.*, self-assessed *versus* clinician-assessed mFG and Hirsuta score versus clinician-assessed mFG). The diagnosis of hirsutism was determined according to cutoff values recommended by the ES and ESHRE. Clinician-assessed mFG scores were considered the gold standard.

## RESULTS

Table 1 shows the characteristics of the participants. The participants had (mean ± standard deviation) age of 33.7 ± 9.9 years, BMI of 29.8 ± 7.21 kg/m^2^, and abdominal circumference of 92.4 ± 15.9 cm. The ethnic composition of the sample was as follows: 21.4% White, 28.7% Black, 47.8% Mixed, and 2.1% of other backgrounds (Indigenous, Asian). The most common comorbidity observed was polycystic ovary syndrome (present in 22.5% of the participants), followed by hypertension (14.9%), diabetes mellitus (13.9%), anxiety (11.9%), congenital adrenal hyperplasia (4.2%), and Cushing’s syndrome (1%).

**Table 1:** Characteristics of the participants (n = 188) categorized according to the Endocrine Society definition of hirsutism for Latin-American women (mFG ≥ 6)

Characteristics	Values
Age (years) – mean ± SD	33.7 ± 9.9
BMI (kg/m^2^) – mean ± SD	29.8 ± 7.21
Abdominal circumference (cm) – mean ± SD	92.4 ± 15.9
Ethnic group – %	
White	23.1
Black	25.8
Mixed	48.97
Other	2.1
Menarche (age) – mean ± SD	12.6 ± 2.14
mFGs – median (min-max)	8.25 (6-16)
Bothered by hirsutism (yes) – %	50
Alopecia (yes) – %	39.1
Oligo/amenorrhea (yes) – %	50.5
Diagnosis – %	
PCOS	23.80
CAH	4.08
Cushing’s syndrome	2.04
Hypertension	12.24
Diabetes mellitus	10.88
Anxiety/depression	15.64
Other	36.73

Abbreviations: BMI, body mass index; CAH, congenital adrenal hyperplasia; mFG, modified Ferriman-Gallwey scoring system; min-max, minimum and maximum values; PCOS, polycystic ovary syndrome.

The correlation value between self-assessed and clinician-assessed mFG scores was 0.478 (95% confidence interval [CI] 0.354-0.588), indicating a moderate level of agreement. The Bland-Altmann analysis identified a bias of -4.67 (95% CI -5.37 to -3.96). The lower limit of agreement was -13.84 (95% CI -15.05 to -12.64) and the upper limit was 4.50 (95% CI 3.30-5.71; p < 0.001), indicating a lack of concordance between the assessments.

**Figure 2:**
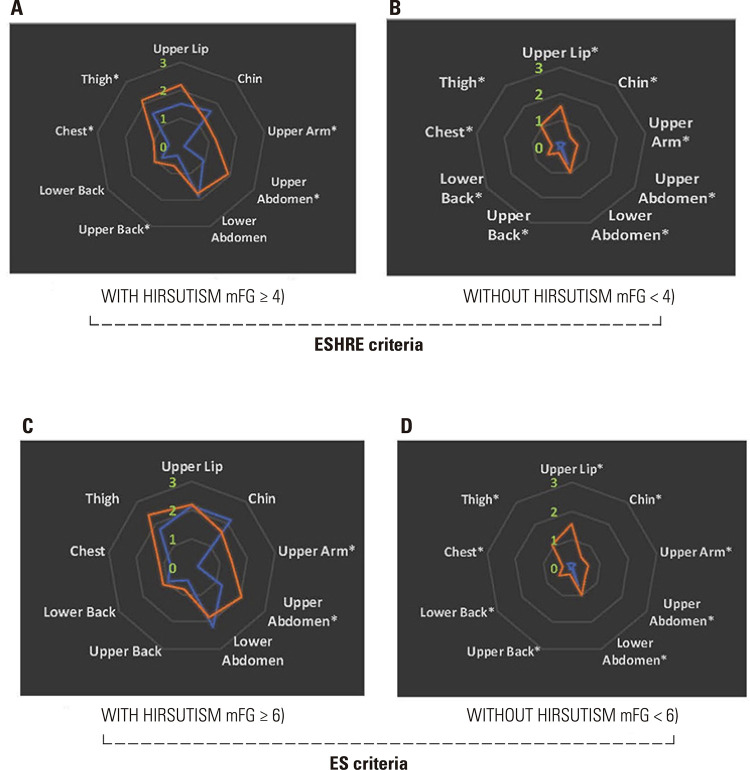
Radar charts comparing self-assessed (orange lines) and clinician-assessed (blue lines) mean scores in each of the nine areas evaluated by the modified Ferriman-Gallwey (mFG) system. The charts are categorized according to the presence or absence of hirsutism (as determined by clinician assessment) and utilizing cutoff values proposed by the (A and B) European Society for Human Reproduction and Embryology (ESHRE) and (C and D) Endocrine Society (ES). The numbers (green) correspond to score values. Abbreviation: mFG, modified Ferriman-Gallwey scoring system. *P < 0.01.

Overall, self-assessed mFG scores were greater than clinician-assessed mFG scores. As shown in Figure 1, self-assessed mFG scores were significantly higher than clinician-assessed scores both in women with and without hirsutism, as determined by ESHRE and ES cutoffs. This trend of overestimation is concerning, as it shows that women categorized as not hirsute by clinician assessment reported self-assessed mFG scores on par with those women who were considered hirsute by clinicians according to the ESHRE criteria (Figure 1A). Importantly, among women who were determined not to have hirsutism based on clinician-assessed scores, the mean self-assessed score fell within the hirsutism cutoff values established by the American Society for Reproductive Medicine (ASRM) and ESHRE. This discrepancy could lead to the misclassification of these women as having hirsutism (Figure 1A and 1B). An analysis by individual areas of body hair revealed significant differences between self-assessed and clinician-assessed scores in the chest, upper arm, upper abdomen, and upper back (according to ESHRE criteria) (Figure 2A) and upper arm and upper abdomen (using ES criteria) (Figure 2C) among women determined to have hirsutism based on clinician-assessed scores (p < 0.05 for all these areas). However, for women classified as not having hirsutism by clinician assessment – regardless of whether ESHRE or ES criteria were applied – the self-assessed scores in all nine areas evaluated were significantly higher than the corresponding clinician-assessed scores (Figures 2B and 2D).

**Figure 3:**
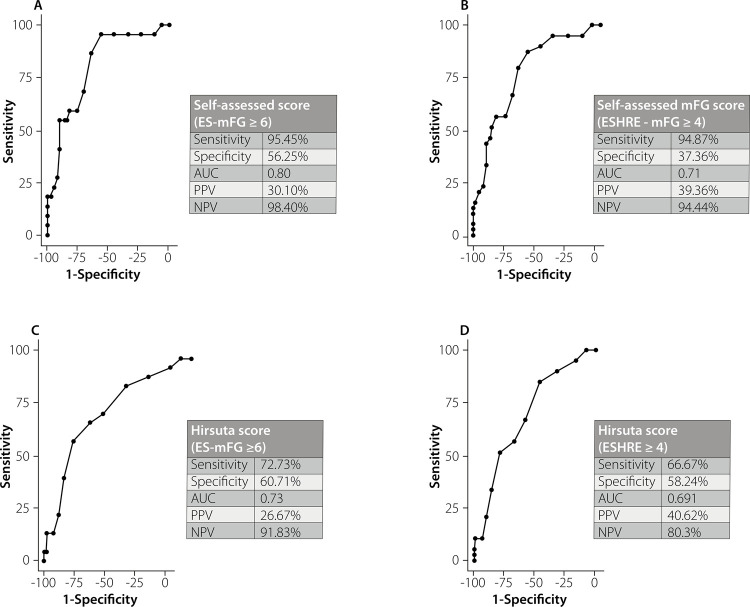
Receiver operating characteristic (ROC) curves of the diagnosis of hirsutism according to self-assessed modified Ferriman-Gallwey scores and Hirsuta scores. The graphs are categorized according to cutoff values proposed by the (A and B) Endocrine Society (ES) and (B and D) European Society for Human Reproduction and Embryology (ESHRE). Abbreviations: AUC, area under the curve; mFG, modified Ferriman-Gallwey; NPV, negative predictive value; PPV, positive predictive value.

Figure 3 shows receiver operating characteristic (ROC) curves of the accuracy (area under curve [AUC]), sensitivity, specificity, positive predictive value (PPV), and negative predictive value (NPV) of self-assessed mFG scores. The best accuracy of the self-assessed mFG score was with the ES criteria for diagnosing hirsutism; in this context, the AUC was 0.80, sensitivity 95.45%, specificity 56.25%, PPV 30.10%, and NPV 98.40% (Figure 3A). However, these metrics declined when the ESHRE criteria was applied for diagnosing hirsutism: AUC of 0.71, sensitivity of 94.87%, specificity 37.36%, PPV 39.36%, and NPV 94.44% (Figure 3B). The Hirsuta score had lower performance compared with the self-assessed mFG score, with AUCs of 0.73 (ES criteria) (Figure 3C) and 0.69 (ESHRE criteria) (Figure 3D).

## DISCUSSION

The mFG score is the mainstay for the diagnosis of hirsutism (3,4). Because the mFG assessment requires specific conditions and examiner training, other alternatives to identify hirsutism based on nonstructured or structured questionnaires have been explored. The present study evaluated the accuracy of a structured questionnaire (self-assessed mFG) in diagnosing hirsutism in a mixed population considering the cutoff values for hirsutism of ≥ 6 as recommended by the ES for Latin-American women and ≥ 4 as recommended by the ESHRE.

The results showed that self-assessed mFG was only moderately accurate in identifying the presence of excessive hair growth and had a critically limiting low specificity. In agreement with the literature, the self-assessed mFG exhibited a high NPV (98.4%), which may make this tool useful for screening the presence of hirsutism in large epidemiological surveys (2,7). Notably, studies have shown that around 39%-69% of women bothered by excess hair identified by self-reported questionnaires do not meet mFG criteria for hirsutism (2,7). The results of the present study identified almost 50% of women with self-assessed mFG scores compatible with hirsutism who were not considered hirsute when evaluated by trained examiners.

Ethnicity affects the cutoff values of mFG scores. In a Brazilian study published a few years ago, a simplified self-assessment questionnaire named Hirsuta, which was based on self-reported mFG scores in five areas (upper lip, chin, chest, lower abdomen, and thighs), reported a diagnostic accuracy for hirsutism of 88.9%, with sensitivity of 85% and specificity of 90%, using a mFG score cutoff value of ≥ 8 (11). The population of that study, aged 35-72 years, was composed mainly of Black women (78.7%), while non-Black participants comprised 21.3% of the sample (11). This sample composition contrasts with that of our sample, which had 28.7% of Black and 71.3% of non-Black participants, all of whom younger than 51 years. Contrary to what we had expected, the Hirsuta score had a lower accuracy (73% and 69%, respectively, according to the latest ES and ESHRE criteria) than the self-assessed mFG score.

Our study has some strengths. First, the clinician-assessed mFG scores were independently rated by two blinded and trained examiners. Second, the sample included a reasonable number of participants with variable degrees of hirsutism. Limitations of the study include the use of a convenience sample of participants identified from an outpatient clinic, many of whom had comorbidities and used medications. By using this type of approach to select the participants, we were unable to analyze the prevalence of hirsutism; therefore, the results observed are based on a potentially higher prevalence rate, which typically reduces the NPV and increases the PPV. Despite of that, the usefulness of a high NPV remains unchanged: in a simulation, the NPV reaches 99.58% when the prevalence of hirsutism is assumed to be 5% and decreases slightly to 98.59% when the prevalence is assumed to be 15%.

In conclusion, this study found limitations in the clinical use of a self-assessed structured questionnaire to establish the diagnosis of hirsutism. However, this type of questionnaire may be useful for screening in epidemiological studies where the exclusion of women with excessive hair growth is intended. Future research using image-based artificial intelligence could enhance the quantification of excessive hair, promoting more reproducible and accurate evaluations.

## Supplemental Figure 1. Questionnaire for self-assessment of the modified Ferriman-Gallwey scoring system.


