# Supplementary material for: Comparison of self-assessed and clinician-assessed hirsutism diagnosed according to the modified Ferriman-Gallwey scale among female outpatients in Brazil
Source: Arch Endocrinol Metab. 2024 Jun 24;68:e230271. doi: 10.20945/2359-4292-2023-0271 (PMC11213573; doi:10.20945/2359-4292-2023-0271)
Supplement: Supplementary file 1 [file 2359-4292-aem-68-e230271-suppl01.pdf]

Supplemental Figure 1. Questionnaire for self-assessment of the modified Ferriman-Gallwey scoring system.

ESCORE DE FERRIMAN: AUTOAVALIAÇÃO

Nome: ____Data:________ Escore Final:______

Este é um questionário que busca identificar se existe aumento de pelos (hirsutismo). Procure responder como você classifica a presença de pelos **(somente os grandes, pigmentados)** nestas 9 regiões do corpo **na data de hoje.** Faça um círculo no desenho que representa melhor o padrão de pelos em você. Caso esteja depilada, por favor nos informe também, incluindo o tempo desde a última depilação, o método e se descoloriu os pelos.

1. LÁBIO SUPERIOR:

( ) **NÃO DEPILADO**

( ) **DEPILADO HÁ ___DIAS**: Método ( ) cera. ( ) lâmina. ( ) laser ( ) outro

- descoloriu algum pelo? ( ) SIM ( )NÃO

Zero:
(nenhum pelo aumentado de tamanho)

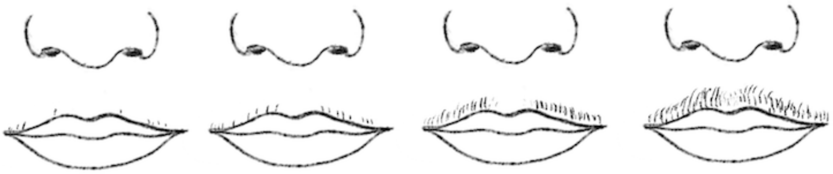

0 1 2 3 4

**ZERO (O): NÃO TENHO NENHUM PELO AUMENTADO**	**1: Alguns pelos grossos isolados (raros)**	**2: vários pelos grossos ocupando parte do lábio**	**3: bigodinho fino**	**4: vários pelos no lábio superior, padrão masculino**

2. QUEIXO (MENTO):

( ) **NÃO DEPILADO**

( ) **DEPILADO HÁ ___DIAS**: Método ( ) cera. ( ) lâmina. ( ) laser ( ) outro

- descoloriu algum pelo? ( ) SIM ( ) NÃO

Zero:
(nenhum pelo aumentado de tamanho)

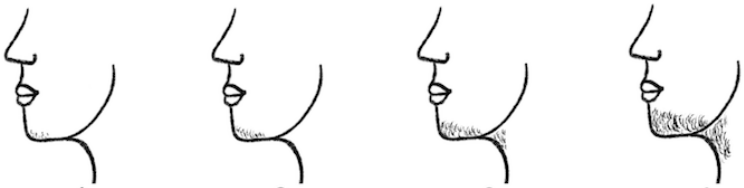

0 1 2 3 4

**ZERO (O): NÃO TENHO NENHUM PELO AUMENTADO**	**1: Alguns pelos grossos isolados (raros) no queixo**	**2: vários pelos grossos ocupando parte do queixo, nos 2 lados**	**3: pequeno cavanhaque**	**4: vários pelos ao longo do queixo padrão masculino**

Pag. 1

Copyright© AE&M all rights reserved.

3. BRAÇO:
( ) **NÃO DEPILADO**
( ) **DEPILADO HÁ ___DIAS**: Método ( ) cera. ( ) lâmina. ( ) laser ( ) outro
- descoloriu algum pelo? ( ) SIM ( )NÃO

Zero:
(nenhum pelo aumentado de tamanho)
0

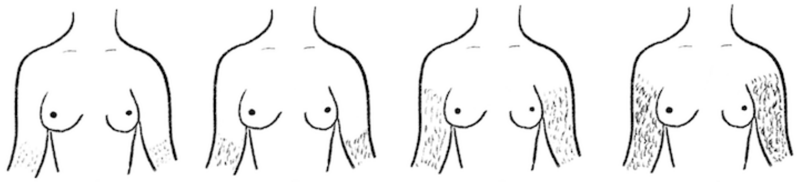

1 **2** **3** **4**

**ZERO (O): NÃO TENHO NENHUM PELO AUMENTADO**	**1: Um ou alguns pelos grossos e longos isolados (raros) no braço**	**2: vários pelos grossos ocupando parte do braço**	**3: maior quantidade de pelos grossos em quase todo o braço, mas com falhas**	**4: braço totalmente coberto de pelos grossos e longos**

4. TÓRAX
( ) **NÃO DEPILADO**
( ) **DEPILADO HÁ ___DIAS**: Método ( ) cera. ( ) lâmina. ( ) laser ( ) outro
- descoloriu algum pelo? ( ) SIM ( )NÃO

Zero:
(nenhum pelo aumentado de tamanho)

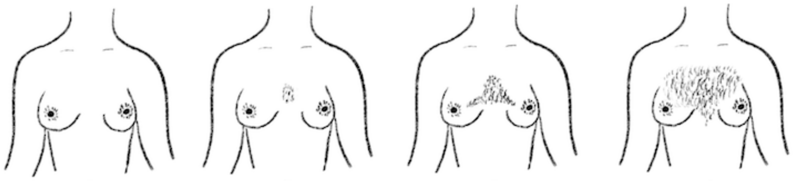

0 **1** **2** **3** **4**

**ZERO (O): NÃO TENHO NENHUM PELO AUMENTADO**	**1: Um ou alguns pelos grossos isolados (raros) em 1 só lugar (ou ao redor do mamilo ou entre as mamas)**	**2: vários pelos grossos ocupando mais de uma região do tórax (ao redor do mamilo e entre as mamas)**	**3: área maior de pelos grossos juntando mais de uma região, mas com falhas**	**4: vários pelos ao longo de todo o peito com padrão masculino**

5. ABDÔMEN SUPERIOR
( ) **NÃO DEPILADO**
( ) **DEPILADO HÁ ___DIAS**: Método ( ) cera. ( ) lâmina. ( ) laser ( ) outro
- descoloriu algum pelo? ( ) SIM ( ) NÃO

Zero:
(nenhum pelo aumentado de tamanho)

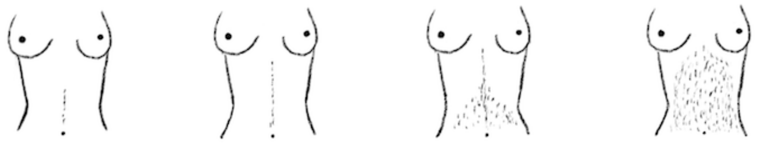

0 **1** **2** **3** **4**

**ZERO (O) : NÃO TENHO NENHUM PELO AUMENTADO**	**1: Um ou mais pelos grossos isolados (raros) acima do umbigo**	**2: vários pelos grossos fazendo um "carreirinho" acima do umbigo**	**3: área maior de pelos grossos se expandido para os lados do abdome**	**4: vários pelos ao longo de todo o abdômen com padrão masculino**

Pag. 2

Copyright© AE&M all rights reserved.

6. ABDÔMEN INFERIOR:

( ) **NÃO DEPILADO**

( ) **DEPILADO HÁ ___DIAS**: Método ( ) cera. ( ) lâmina. ( ) laser ( ) outro

- descoloriu algum pelo? ( ) SIM ( ) NÃO

Zero:
(nenhum pelo aumentado de tamanho

0

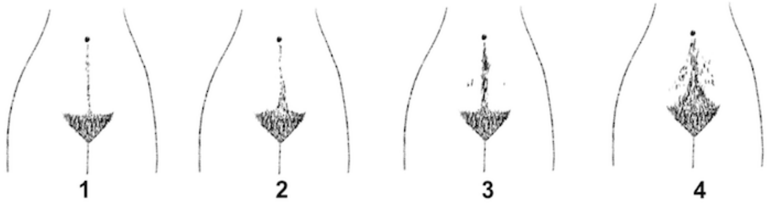

**ZERO (O): NÃO TENHO NENHUM PELO AUMENTADO**	**1: Um ou alguns pelos grossos abaixo do umbigo**	**2: vários pelos grossos em sequência, fazendo um "carreirinho" abaixo do umbigo**	**3: área maior de pelos grossos se expandido para os lados do abdome (abaixo do umbigo)**	**4: abdômen inferior totalmente coberto de pelos grossos e longos**

7. DORSO

( ) **NÃO DEPILADO**

( ) **DEPILADO HÁ ___DIAS**: Método ( ) cera. ( ) lâmina. ( ) laser ( ) outro

- descoloriu algum pelo? ( ) SIM ( ) NÃO

Zero:
(nenhum pelo aumentado de tamanho)

0

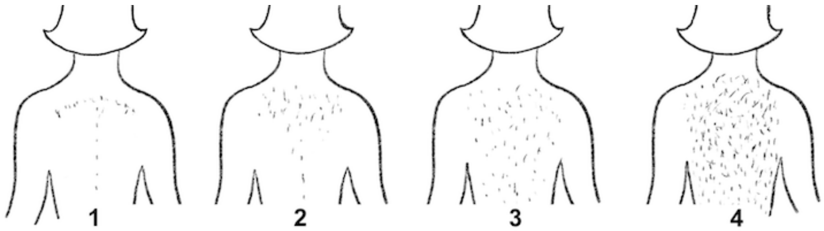

**ZERO (O): NÃO TENHO NENHUM PELO AUMENTADO**	**1: Um ou alguns pelos grossos isolados (raros) no dorso**	**2: vários pelos grossos ocupando mais de uma região do dorso**	**3: dorso quase todo cheio de pelos grossos e longos (mas com falhas)**	**4: vários pelos ao longo de todo o dorso com padrão masculino**

8. LOMBAR

( ) **NÃO DEPILADO**

( ) **DEPILADO HÁ ___DIAS**: Método ( ) cera. ( ) lâmina. ( ) laser ( )outro

- descoloriu algum pelo? ( ) SIM ( ) NÃO

Zero:
(nenhum pelo aumentado de tamanho)

0

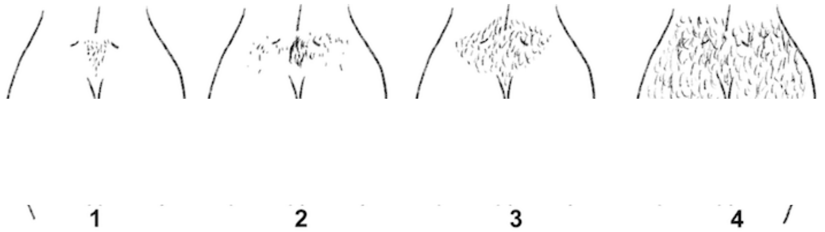

**ZERO (O): NÃO TENHO NENHUM PELO AUMENTADO**	**1: Um ou mais pelos grossos isolados (raros) na região lombar**	**2: vários pelos grossos juntos na região lombar**	**3: área maior de pelos grossos se expandido para os lados da região lombar**	**4: vários pelos ao longo de todo a região lombar com padrão masculino**

Pag. 3

Copyright© AE&M all rights reserved.

9. RAIZ DAS PERNAS

( ) **NÃO DEPILADO**

( ) **DEPILADO HÁ ___DIAS**: Método ( ) cera. ( ) lâmina. ( ) laser ( ) outro

- descoloriu algum pelo? ( ) SIM ( ) NÃO

Zero:
(nenhum pelo aumentado de tamanho

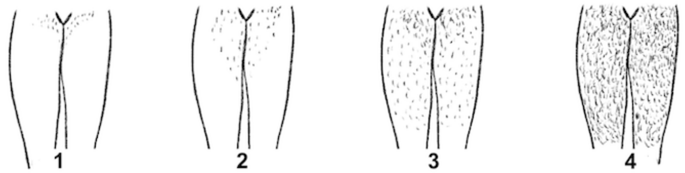

0

**ZERO (O): NÃO TENHO NENHUM PELO AUMENTADO**	**1: Um ou alguns pelos grossos nas coxas**	**2: vários pelos grossos em sequência em mais de uma região ou se estendendo para baixo**	**3: área maior de pelos grossos se expandido para todas as partes da coxa, mas com falhas (áreas abertas)**	**4: coxas totalmente cobertas de pelos grossos e longos**

This is an open-access article distributed under the terms of the Creative Commons Attribution License

Copyright© AE&M all rights reserved.
